# Andrographolide Attenuates Gut-Brain-Axis Associated Pathology in Gulf War Illness by Modulating Bacteriome-Virome Associated Inflammation and Microglia-Neuron Proinflammatory Crosstalk

**DOI:** 10.3390/brainsci11070905

**Published:** 2021-07-09

**Authors:** Punnag Saha, Peter T. Skidmore, LaRinda A. Holland, Ayan Mondal, Dipro Bose, Ratanesh K. Seth, Kimberly Sullivan, Patricia A. Janulewicz, Ronnie Horner, Nancy Klimas, Mitzi Nagarkatti, Prakash Nagarkatti, Efrem S. Lim, Saurabh Chatterjee

**Affiliations:** 1 Environmental Health and Disease Laboratory, Department of Environmental Health Sciences, University of South Carolina, Columbia, SC 29208, USA; psaha@email.sc.edu (P.S.); mondala@mailbox.sc.edu (A.M.); bosed@email.sc.edu (D.B.); sethr@mailbox.sc.edu (R.K.S.); 2 Center for Fundamental and Applied Microbiomics, The Biodesign Institute, Arizona State University, Tempe, AZ 85281, USA; ptskidmo@asu.edu (P.T.S.); LaRinda.Holland@asu.edu (L.A.H.); Efrem.Lim@asu.edu (E.S.L.); 3 Department of Environmental Health, Boston University School of Public Health, Boston, MA 02118, USA; tty@bu.edu (K.S.); paj@bu.edu (P.A.J.); 4 College of Public Health, University of Nebraska Medical Center, Omaha, NE 68198, USA; rhorner@unmc.edu; 5 Institute for Neuro-Immune Medicine, Nova Southeastern University, Fort Lauderdale, FL 33314, USA; nklimas@nova.edu; 6 Department of Pathology, Microbiology and Immunology, University of South Carolina School of Medicine, Columbia, SC 29209, USA; mitzi.nagarkatti@uscmed.sc.edu (M.N.); PRAKASH@mailbox.sc.edu (P.N.); 7 School of Life Sciences, Arizona State University, Tempe, AZ 85281, USA; 8 Columbia VA Medical Center, Columbia, SC 29209, USA

**Keywords:** Gulf War Illness, andrographolide, virome, dysbiosis, inflammation, IL-6, Tau, BDNF, microglia

## Abstract

Gulf War Illness (GWI) is a chronic multi-symptomatic illness that is associated with fatigue, pain, cognitive deficits, and gastrointestinal disturbances and presents a significant challenge to treat in clinics. Our previous studies show a role of an altered Gut–Brain axis pathology in disease development and symptom persistence in GWI. The present study utilizes a mouse model of GWI to study the role of a labdane diterpenoid andrographolide (AG) to attenuate the Gut–Brain axis-linked pathology. Results showed that AG treatment in mice (100 mg/kg) via oral gavage restored bacteriome alterations, significantly increased probiotic bacteria *Akkermansia*, *Lachnospiraceae*, and *Bifidobacterium*, the genera that are known to aid in preserving gut and immune health. AG also corrected an altered virome with significant decreases in virome families *Siphoviridae* and *Myoviridae* known to be associated with gastrointestinal pathology. AG treatment significantly restored tight junction proteins that correlated well with decreased intestinal proinflammatory mediators IL-1β and IL-6 release. AG treatment could restore Claudin-5 levels, crucial for maintaining the BBB integrity. Notably, AG could decrease microglial activation and increase neurotrophic factor BDNF, the key to neurogenesis. Mechanistically, microglial conditioned medium generated from IL-6 stimulation with or without AG in a concentration similar to circulating levels found in the GWI mouse model and co-incubated with neuronal cells in vitro, decreased Tau phosphorylation and neuronal apoptosis. In conclusion, we show that AG treatment mitigated the Gut–Brain-Axis associated pathology in GWI and may be considered as a potential therapeutic avenue for the much-needed bench to bedside strategies in GWI.

## 1. Introduction

Gulf War Illness (GWI) is a chronic, multi-symptomatic, and medically unexplained disorder that affected almost a third of the returning veterans deployed in the Persian Gulf War of 1990–91 [1,2]. As per the epidemiological study conducted by Steele et al. on a large population of Kansas Gulf War veterans, reported cases of GWI should enlist at least three moderate to severe symptoms out of six pre-defined symptom domains to be qualified as GWI [3]. These pre-defined six domains consist of i. chronic fatigue problems, ii. somatic pain, iii. neurological or cognitive problems, iv. gastrointestinal (GI) problems, v. respiratory problems, vi. dermatological issues. The deployed veterans were directly exposed to numerous toxicants during the wartime, including pesticides such as permethrin (Per), nerve gas antidote pyridostigmine bromide (PB), chemical agents, e.g., sarin gas, mustard gas, depleted uranium, as well as various combustion products from the burnt oil wells [4,5]. Exposure to these hazardous chemicals combined with wartime stress resulted in the persistence of the prolonged symptoms among the veterans even after 30 years since the war ended. Understanding and treating the myriad of elusive symptoms associated with GWI etiology remains a major challenge for both clinicians and scientists. 

We were the first group to establish a notable connection between the gut microbiome and GWI pathology, where we showed that the administration of Gulf War chemicals (GWC) in a mice model caused an alteration of the gut bacteriome signature, which in turn led to increased intestinal inflammation, gut leaching, endotoxemia, and release of the damage-associated molecular pattern (DAMP) HMGB1 (High motility group box 1) [6,7]. Subsequently, we have shown that GWI-associated dysbiosis in mice resulted in a decrease of the probiotic butyrogenic bacteria [7,8], increased *Firmicutes* abundance over *Bacteroidetes* in the phylum level [6,7], and a decrease of the probiotic species *Akkermansia muciniphila* [9] and these alterations attributed to increased intestinal inflammation, gut leaching [7,8,10,11], enteric glial cell activation [12], hepatic metabolic reprogramming [8,11], neuronal inflammation [6,7,9,10], the systemic release of HMGB1 [7,8], symptoms all of which fall under GWI pathology domain. We have also shown that administration of oral butyrate and the nutraceutical Sparstolonin B in mice helped to restore normal bacteriome patterns and decreased GWI-associated symptoms as described previously. In addition to bacteriome alteration, we were the first group to report that GWC treatment in mice changed the gut virome pattern, where we observed an increased abundance of *Siphoviridae* and *Myoviridae* along with decreases in *Microviridae* abundance [10]. This virome dysbiosis correlated with GWI pathological symptoms, and the use of the anti-viral drug Ribavirin helped to diminish the symptoms [10]. These studies formed the basis of our microbiome targeted therapy for GWI, as the lack of proper therapeutic compounds is preventing affected veterans from getting treatment.

Andrographolide (AG) is a well-known bioactive phytoconstituent extracted from the plant *Andrographis paniculata* and is highly popular as an alternative medicine in South-East Asian countries, e.g., India, China. Structure wise, AG is a labdane diterpenoid, which exerts numerous pharmacological effects including anti-inflammation [13,14], anti-oxidative stress [15], anti-cancer [16], hepatoprotection [17,18], anti-fibrosis [19,20], and neuroprotection [21,22,23]. Mechanistically, AG is a known inhibitor of the nuclear factor kappa-light-chain-enhancer of activated B cells (NF-kB) pathway, which explains its anti-inflammatory nature [24]. Besides inhibiting the NF-kB pathway, AG has been shown to participate in various signaling pathways in various cell types and organ systems [25]. Interestingly, Bera et al. showed that AG can cross the blood–brain barrier [26] and possibly can enter into the cells via passive diffusion, as any AG-specific receptor has not been found to date [27]. Considering all the beneficial roles that AG possesses and its multi-target nature, we wanted to observe whether AG administration in GWC treated mice can restore their altered bacteriome as well as the virome pattern and attenuate GWI-associated symptoms, including gut inflammation, leaking of the gut, systemic inflammation, and various neuropathology. We also wanted to determine the role of AG in diminishing Interleukin-6 (IL-6) treated microglial activation and resulting neuronal apoptosis in vitro.

## 2. Materials and Methods

### 2.1. Materials

Permethrin (PER), pyridostigmine bromide (PB) were obtained from Sigma-Aldrich (St. Louis, MO, USA), whereas andrographolide (AG) was bought from Cayman Chemical (Ann Arbor, MI, USA). Primary antibodies, including anti-Occludin, anti-Claudin-2, and anti-transmembrane protein 119 (TMEM119), anti-phosphorylated Tau (p-Tau) were purchased from Abcam (Cambridge, MA, USA). Anti-Flotillin 1, anti-interleukin-1β (IL-1β), anti-interleukin-6 (IL-6), anti-brain-derived neurotrophic factor (BDNF), and anti-Cluster of Differentiation 40 (CD40) primary antibodies were bought from Santacruz Biotechnology (Dallas, TX, USA). Anti-myeloid differentiation primary response protein (MyD88), anti-Toll-like receptor 4 (TLR4) primary antibodies were purchased from Abclonal Technology (Woburn, MA, USA), whereas Anti-NF-κB p65, anti-phospho NF-κB p65, anti-Tau, anti-Bcl-2-associated X protein (BAX), anti-Cleaved Caspase 8, anti-Caspase 8, anti-Cleaved Caspase 3, Caspase 3 antibodies were purchased from Cell Signaling Technology (Danvers, MA, USA). Anti-β-actin and anti-B-cell lymphoma 2 (Bcl-2) antibodies were purchased from Proteintech (Rosemont, IL, USA). Species-specific biotinylated conjugated secondary antibody and Streptavidin-horse radish peroxidase (HRP) (Vectastain Elite ABC kit) were obtained from Vector laboratories (Burlingame, CA, USA). Fluorescence conjugated Alexa Fluor secondary antibodies, as well as ProLong Diamond antifade mounting media with 4′,6-diamidino-2-phenylindole (DAPI), were bought from Thermo Fisher Scientific (Rockford, IL, USA). Recombinant mouse IL-6 was purchased from Sino Biological (Wayne, PA, USA). All the necessary chemicals used in this study were purchased from Sigma-Aldrich (St. Louis, MO, USA). Murine small intestine and brain tissues were processed at AML Laboratories (Jacksonville, FL, USA) and Instrument Resources Facility, University of South Carolina School of Medicine (Columbia, SC, USA) for paraffin-embedding and sectioning into slides. Bacteriome analysis was performed by Cosmos ID (Rockville, MD, USA).

### 2.2. Animals

Adult (10 weeks old), wild-type, pathogen-free, male C57BL/6J mice were purchased from Jackson Laboratories (Bar Harbour, ME, USA). Mice experiments were conducted maintaining the National Institutes of Health (NIH) guideline for human care and use of laboratory animals and local Institutional Animal Care and Use Committee (IACUC) standards (Animal Use Protocol reference number: 101345; Protocol Number: 2419-101345-072318; approval date: 23 July 2020). The animal handling procedures were approved by the University of South Carolina (Columbia, SC, USA). Upon arrival, all mice were housed in a temperature (22–24 °C) and humidity-controlled animal room and had ad libitum access to chow diet and water with a 12-h light/12-h dark cycle. Mice were sacrificed after completion of the dosage regimen. Immediately after anesthetization, blood was collected from mice using the cardiac puncture method, and serum was extracted from it. All serum samples were preserved at −80 °C until further analysis was performed. Organs, including the distal parts of the small intestine, were collected and fixed in 10% neutral buffered formaldehyde (Sigma-Aldrich, St. Louis, MO, USA), whereas the frontal cortexes were collected and fixed in Bouin’s solution (Sigma-Aldrich, St. Louis, MO, USA). Fecal pellets were collected from the colon and preserved at −80 °C for microbiome analysis.

### 2.3. Mouse Model of Gulf War Illness

GW chemicals (PER and PB) were used to expose the mice following well-established acute rodent models of Gulf War illness with some modifications [7,9,11]. All mice were randomly divided into 4 groups after 1 week of acclimatization. The 1st group was CONTROL (*n* = 11), and mice were administered with vehicle (0.6% DMSO diluted in PBS) only. The 2nd group was GWT (*n* = 11), where mice were administered with the mixture of GW chemicals PER (200 mg/kg body weight, dissolved in DMSO and diluted in PBS; the final concentration of DMSO was 0.6%) and PB (2 mg/kg body weight; diluted in PBS) by oral gavage. The 3rd group (GWT+AG) (*n* = 6) was treated with both GW chemicals and AG. Mice were dosed with GW chemicals first, followed by AG (100 mg/kg body weight) by oral gavage. Finally, the 4th group of mice received only andrographolide (AG) (*n* = 6) by oral gavage, similar to the 3rd group. GW chemicals and AG were administered to the mice every alternate day for a 1-week time period. Each mouse, according to their assigned group, received 100 µL of the vehicle or GW chemicals and/or AG.

### 2.4. Cell Culture

#### 2.4.1. Mouse Microglial Cell Culture and Treatment

Immortalized mouse microglial SIM-A9 cell line (ATCC^®^ CRL-3265™) was purchased from ATCC (Manassas, VA, USA). SIM-A9 cells were maintained using a complete growth medium consisting of Dulbecco’s Modified Eagle Medium: F12 (DMEM: F-12 Medium) (ATCC^®^ 30-2006™) supplemented with 10% fetal bovine serum (FBS), 5% heat-inactivated horse serum, 100 U/mL Penicillin, 100 µg/mL Streptomycin (Gibco, NY, USA) and incubated in a humidified 5% CO_2_ incubator at 37 °C. Prior to cell passage, SIM-A9 cells were washed with Dulbecco’s phosphate-buffered saline (DPBS) and then detached using a mixture of 1mM EDTA, 1 mM EGTA, and 1 mg/mL glucose in DPBS. 

Approximately 0.3 × 10^6^ cells/well were seeded onto a 6 well tissue culture plate. Serum starvation was performed using DMEM: F-12 Medium containing 1% FBS only for 18 h when the cells attained almost 70% confluency. Following that, cells were treated with vehicle control (0.05% DMSO) only (CONTROL), mouse recombinant IL-6 (64 pg/mL) only (IL-6), 50 µM andrographolide only (AG50), and a combination of both IL-6 (64 pg/mL) and 50 µM andrographolide (IL-6+AG50) for 24 h. IL-6 concentration used for this cell treatment was determined by our in vivo IL-6 serum ELISA result. Upon completion of treatment, supernatant from each experimental group was collected, centrifuged, and stored at −80 °C. These supernatants containing the secretome of the treated SIM-A9 cells were used as “Microglial-conditioned Medium” (MCM) to treat the Neuro-2a cells for the next part of our in vitro experiments. In addition, ELISA was performed using these cell supernatants to quantify the level of TNFα secretion by SIM-A9 cells for each experimental group.

#### 2.4.2. Mouse Neuronal Cell Culture and Treatment

Mouse neuroblastoma cell line Neuro-2a (N2a) (ATCC^®^ CCL-131™) was obtained from ATCC (Manassas, VA, USA). These N2a cells were maintained on Eagle’s Minimum Essential Medium (EMEM) (ATCC^®^ 30-2003™) supplemented with 10% FBS, 100 U/mL Penicillin, 100 µg/mL Streptomycin (Gibco, NY, USA) and incubated in a humidified 5% CO_2_ incubator at 37 °C. 

Cells were plated on a 6 well plate with a seeding density of 0.3 × 10^6^ cells/well and were grown till the cells reached almost 70% confluency. Then, the N2a cells were serum-starved for 18 h using EMEM supplemented with 1% FBS. Following serum starvation, the cells were treated with a combination of MCM obtained from each SIM-A9 microglia experimental group and normal N2a growth medium for 24 h. The groups designed for this experiment were unchanged from the last SIM-A9 cells experiment (CONTROL, IL-6, AG50, and IL-6+AG50). Post-treatment, cell supernatants were collected, stored at −80 °C, and proteins from each group were extracted using 1× RIPA lysis buffer containing protease and phosphatase inhibitors. The concentration of the extracted protein samples from each group was estimated using the BCA assay kit (Thermo Fisher Scientific, Rockford, IL, USA), and the Western Blot experiment was performed using these extracted protein samples.

### 2.5. Microbiome Analysis

#### 2.5.1. Bacteriome Analysis

Bacteriome analysis was performed by CosmosID (Rockville, MD, USA) using the fecal pellets obtained from individual mouse samples after euthanization. Briefly, DNA isolation was performed from these fecal pellets using the ZymoBIOMICS Miniprep kit following the manufacturer’s protocol. After that, sequencing was conducted with a 2-step polymerase chain reaction (PCR) targeting the V3V4 (341 nt–805 nt) region of the bacterial 16S ribosomal RNA (rRNA) gene. At first, a 16S-optimized primer was used in PCR to amplify the V3-V4 regions of the 16S ribosomal DNA (rDNA). Then, these PCR amplicons were used as templates with adapters containing 8 bp index for the 2nd step to prepare Illumina dual-index libraries and further processed for sequencing purposes. These dual-index library amplicon products were purified by using Ampure beads (Beckman Coulter, Brea, CA, USA). Quantification of the dual-index library was performed by using Qubit dsDNA HS assay (Thermo Fisher), followed by qualification on a 2100 Bioanalyzer instrument (Agilent, Santa Clara, CA, USA) where the distribution with a peak in the expected range was scrutinized. Final quantification was conducted by performing quantitative PCR (qPCR), followed by loading onto MiSeq (Illumina, San Diego, CA, USA) sequencer for PE250 (v2 chemistry). Sequences obtained for each sample were finally run on the 16S pipeline of the CosmosID GENIUS software, and results were analyzed. This whole bacteriome analysis process for this study was performed according to standard protocol optimized by the vendor.

#### 2.5.2. Virus-Like Particle (VLP) Enrichment and Total Nucleic Acid Extraction

Mouse fecal specimens (approximately 58 mg) were diluted in 1200 µL of SM Buffer (G-Biosciences, Maryland Heights, MO, USA), vortexed at 3000 rpm for 5 min, then centrifuged at 7000× *rcf* for 10 min. The supernatant was filtered through a 0.45 μm membrane (Celltreat, Pepperell, MA, USA). The stool filtrates were treated with lysozyme (Sigma) and baseline-ZERO DNase (Lucigen, Middleton, WI, USA) to degrade unencapsulated DNA. DNase treatment was inactivated with EDTA (5 mM final concentration). Total nucleic acid was extracted using the eMAG instrument (bioMérieux, Marcy-l’Étoile, France). Negative controls were included in the same extraction process to assess contamination. VLP DNA was amplified with GenomiPhi V2 (GE Healthcare, Chicago, IL, USA) before Illumina library construction. The negative controls were spiked with Enterobacteria phage λ DNA to assess cross-contamination and amplification. Samples and negative controls were pooled for sequencing in an Illumina NovaSeq 6000 2 × 150 bp sequencing run. A total of 549,782,556 sequencing reads were generated, resulting in an average of 14,858,988 reads per sample.

Reads with a minimum length of 75 and an average quality score above 20 were retained for further analysis using bbduk. After quality control, 352,385,124 reads remained, resulting in an average of 9,523,922 reads per sample. Viral contigs were assembled using metaSPAdes version 3.14.0 from QC reads (28,298,430). Contigs were then filtered to a minimum length of 500 base pairs using CD-HIT and de-duplicated to a minimum identity of 99% [28,29]. Contigs mapping to the host mouse genome were discarded. Bacteriophage contigs were identified by merging VirSorter called contigs and contigs identified by MegaBLAST as being from bacteriophage families [30]. Eukaryotic contigs were identified by querying contigs against a viral NR database using blastx. Bacteriophage and eukaryotic contigs were then merged, and taxonomy was retrieved using a taxonomizr (Supplementary Table S1). BWA-MEM was used to get the number of reads associated with each contig by mapping the QC reads against the contigs generated. Read counts were then normalized by reads per kilobase hundred thousand in an RPKM fashion. Contaminants were removed using decontam using the prevalence method with a threshold of 0.3 [31]. Decontam identified and removed 14 contaminants leaving an average of 99.98% of viral counts (Supplementary Figure S2). Richness, Shannon diversity, and beta diversity measurements were calculated with the QIIME2 package [32]. Discriminant analysis was performed using LEfSe to determine features between treatment groups and MaaAsLin2 for discriminant features by cytokine and protein levels [33]. Data visualization and statistical tests were conducted using Graphpad Prism. All virome comparisons were conducted using the Mann–Whitney U test.

#### 2.5.3. Availability of Data and Materials

Sequence data are in the processing of being deposited to the NCBI Sequence Read Archive under BioProject (accession number PRJNA731934).

### 2.6. Laboratory Methods

#### 2.6.1. Immunohistochemistry

Deparaffinization procedure of paraffin-embedded small intestine and brain tissue sections was performed following standard laboratory procedure. Briefly, 5 μm-thick tissues were immersed successively in 100% xylene, 1:1 solution of xylene and ethanol, 100% ethanol, 95% ethanol, 70% ethanol, 50% ethanol, deionized water for 3 min each. Following deparaffinization, antigen epitope retrieval was conducted by using the Epitope retrieval solution and steamer (IHC-World, Woodstock, MD, USA). Endogenous peroxidase activity was blocked by using 3% H_2_O_2_ solution for 20 min, followed by serum blocking (5% goat serum) for 1 h. After serum blocking, primary antibodies for IL-1β, IL-6, and BDNF were diluted (1:300) in blocking buffer and applied to the tissue sections. All sections were kept at 4 °C for overnight incubation in a humidified chamber. After overnight incubation, the tissue sections were washed with 1X PBS-T (PBS+ 0.05% Tween 20) 3 times. Biotinylated secondary antibodies (species-specific) were probed at 1:250 dilution, followed by incubation with streptavidin-conjugated with horseradish peroxidase at 1:200 dilution. Finally, the chromogenic substrate solution of 3,3 diaminobenzidine (DAB) (Abcam; Cambridge, MA, USA) was applied to the sections, and counterstaining was performed by using Mayer’s hematoxylin (Sigma-Aldrich, St. Louis, MO, USA). Mounting of all tissue sections was conducted by using Simpo mount (GBI Laboratories, Mukilteo, WA, USA). Reactivity of the applied antibodies in the sections was observed under 10× and 20× objectives and images were captured using an Olympus BX43 microscope (Olympus, America). Morphometric data analyses were performed by CellSens Software from Olympus America (Center Valley, PA, USA).

#### 2.6.2. Immunofluorescence Staining

Deparaffinization and epitope retrieval procedures of the paraffin-embedded small intestine and brain tissue sections were performed similarly as mentioned above. After completion of the epitope retrieval process, the tissue sections were permeabilized by using PBS-T (PBS+ 0.1% Triton X-100) solution for 1 h, followed by blocking with 5% goat serum for 1 h. After that, tissue sections were incubated with primary antibodies of anti-Occludin, anti-Claudin-2, anti-TLR4, anti-Flotillin1, anti-Claudin-5, anti-CD31, anti-CD40, and anti-TMEM119 (at 1:300 dilution) and kept overnight at 4 °C in a humidified chamber. Then, species-specific anti-IgG secondary antibodies conjugated with Alexa Fluor 488 or 633 from Invitrogen (Rockford, IL, USA) were used at 1:250 dilutions. Finally, mounting was performed by using ProLong Gold antifade reagent with DAPI (Life Technologies, Carlsbad, CA, USA). Images were taken under 10×, 40×, and 60× magnification with an Olympus BX63 microscope. Morphometric data analyses were performed by CellSens Software from Olympus America (Center Valley, PA, USA).

#### 2.6.3. Western Blot

Proteins were extracted from the small intestine tissue samples and cells using 1× RIPA lysis buffer containing protease and phosphatase inhibitors. Estimation of the extracted tissue and cellular proteins was performed by using the BCA assay kit (Thermo Fisher Scientific, Rockford, IL, USA). Approximately 30 µg of extracted proteins from each sample were added to a mixture containing 1× NuPAGE™ LDS Sample Buffer (Thermo Fisher Scientific, Rockford, IL, USA) and 10% β-mercaptoethanol, followed by boiling for 5 min. Then, the protein samples were subjected to standard SDS-PAGE using Novex 4–12% bis-tris gradient gel. Membrane transfer of separated protein bands on a nitrocellulose membrane was performed by using the Trans-Blot Turbo transfer system (Bio-rad, Hercules, CA, USA), and the membrane was blocked with 3% bovine serum albumin (BSA) for 1 h. Anti-TLR4, anti-TLR7, anti-MyD88, anti-β-actin, anti-NF-κB p65, anti-phospho NF-κB p65, anti-phospho-Tau, anti-total-Tau, anti-BAX, anti-Bcl-2, anti-Cleaved Caspase 8, anti-Caspase 8, anti-Cleaved Caspase 3, anti-Caspase 3 primary antibodies were incubated using 1:1000 dilution overnight at 4 °C. Compatible horseradish peroxidase-conjugated species-specific secondary antibodies were used to probe the primary antibody. Pierce ECL Western blotting substrate (Thermo Fisher Scientific, Waltham, MA, USA) was used for blot development. Finally, the blots were captured by G: Box Chemi XX6, and densitometry analysis was performed using Image J software.

#### 2.6.4. Enzyme-Linked Immunosorbent Assay (ELISA)

Serum IL-1β and IL-6 levels were estimated with serum samples obtained from CONTROL, GWT, GWT + AG, and AG mice groups using the ELISA kit from ProteinTech (Rosemont, IL, USA) following the manufacturer’s protocol. In addition, TNFα ELISA was performed as per the manufacture’s instruction (ProteinTech, Rosemont, IL, USA) to measure the secreted level of TNFα using the mouse microglial SIM-A9 cell supernatants.

### 2.7. Statistical Analyses

All statistical analyses were performed using GraphPad Prism software (San Diego, CA, USA). Unpaired *t*-test (two-tailed tests with equal variance) and one-way analysis of variance (ANOVA) were carried out for determining the difference between multiple groups, followed by Bonferroni–Dunn post-hoc corrections analysis for all intergroup comparisons. For all analyses, *p* ≤ 0.05 was considered statistically significant, and data were presented as the mean ± SEM. 

## 3. Results

### 3.1. Andrographolide Treatment Restores GW Chemical-Associated Bacteriome Alteration in Mice

**Figure 1:**
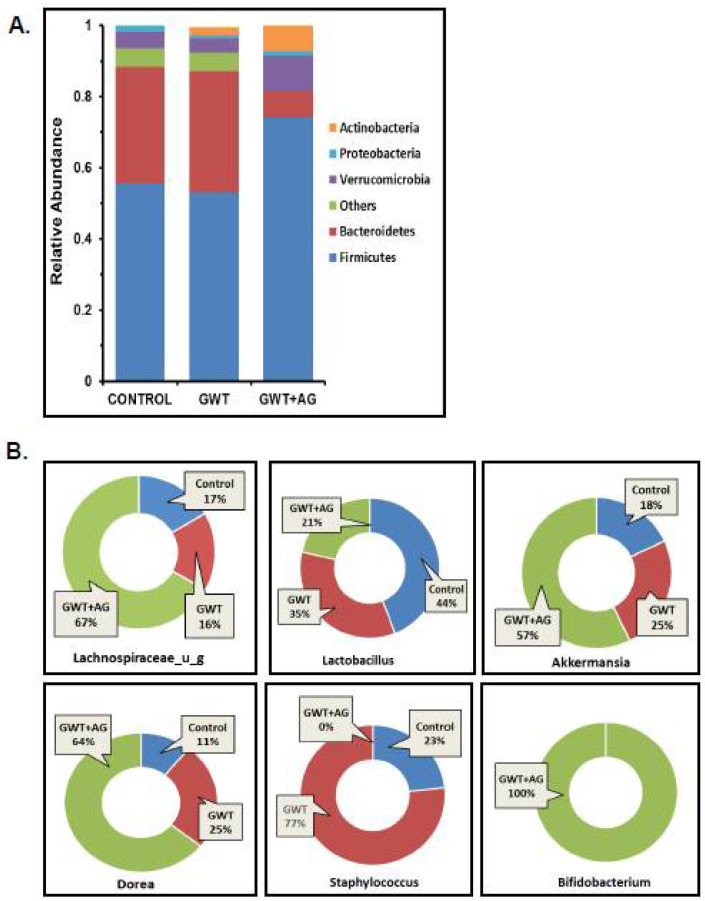
Gulf War (GW) chemical exposure results in alteration of mouse gut bacteriome, while andrographolide (AG) treatment helps in restoration of normal microbiome. (**A**) The relative abundance of the bacteriome is presented by group average at the phylum level for CONTROL (mice treated with vehicle only), GWT (GW chemical exposed mice), and GWT+AG (mice co-exposed to both GW chemicals and AG) groups. (**B**) Graphical representation of six significantly altered bacteria at the genus level. The percentage abundance of (i) *Lachnospiraceae_u_g*, (ii) *Lactobacillus*, (iii) *Akkermansia*, (iv) *Dorea*, (v) *Staphylococcus*, (vi) *Bifidobacterium* has been calculated and presented as the mean for CONTROL, GWT, and GWT+AG groups.

Previous studies from our group have consistently shown that GW chemical administration in mice causes a significant alteration in the normal gut microflora [7,8,9,10,12]. In this study, we wanted to observe whether AG administration can directly affect the dysbiosis of the bacteriome, caused by GW chemicals PB and PER. Using fecal pellets obtained from the mice model of GWI, next-generation sequencing was carried out for detailed microbiome analysis. Consistent with our previous findings, an increase in the relative abundance of the phylum Bacteroidetes was observed parallelly to a decreased relative abundance of Firmicutes in the GWT group (Figure 1A). Conversely, AG treatment significantly increased Firmicutes abundance and decreased Bacteroidetes abundance in the GWT+AG group when compared to the GWT group (Figure 1A). At the genus level, we observed that AG enhanced the abundance of many bacteria, which were beneficial for the host. The relative abundance of the individual genus, calculated between different treatment groups, was depicted as a percentage value. Results showed that *Lachnospiraceae_u_g* and *Akkermansia,* which impart several advantageous effects, including anti-inflammatory properties and mucin layer protection, were increased in the GWT+AG group compared to both Control and GWT groups, respectively (Figure 1B) [34,35]. *Bifidobacterium* is a butyrogenic bacteria that belong to the phylum Firmicutes and is a known probiotic [36]. It was observed that AG treatment via oral gavage administration aided in the relative abundance of *Bifidobacterium* in the intestinal microenvironment of GWT+AG mice, which was otherwise absent in both the Control and GWT groups (Figure 1B). Members of the *Staphylococcus* genus primarily consisted of many opportunistic pathogens [37]. Our analysis showed an increased relative abundance of *Staphylococcus* genus in the GWT group compared to the Control group. Surprisingly, AG treatment led to a total depletion of this genus in the GWT+AG group. However, the relative abundance of *Dorea* was not decreased in the GWT+AG group when compared to the GWT group. In addition, the relative abundance of *Lactobacillus* was not found to be significantly increased in the GWT+AG group, compared to both Control and GWT groups.

### 3.2. Virome Alteration Associated with Andrographolide Administration in a Mouse Model of GWI

**Figure 2:**
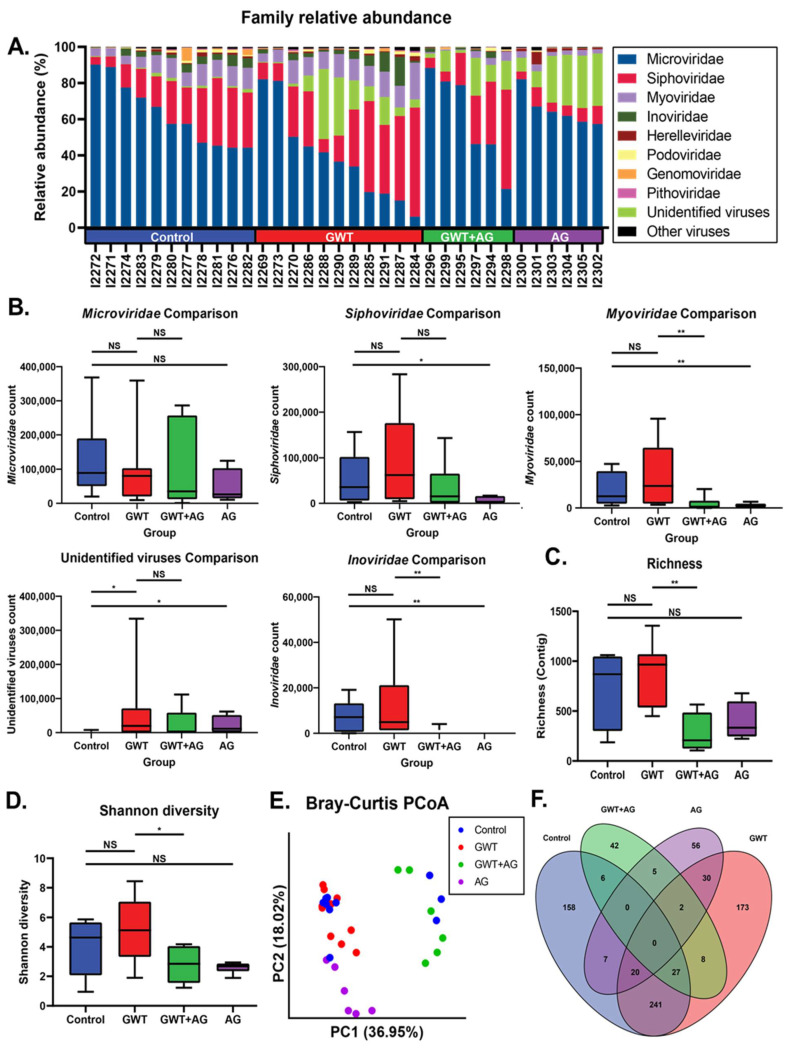
Virome composition and analysis of mice under different GW chemical exposure conditions. (**A**) Relative abundance of most abundant viral families for all mice in the cohort. (**B**) Boxplots of *Microviridae, Siphoviridae, Myoviridae,* and *Inoviridae* NGS RPK counts between treatment groups. Statistical significance was assessed by a Mann–Whitney U test (* *p* < 0.05, ** *p* < 0.01, NS: non-significant). (**C**) Boxplot of viral contig richness between treatment groups. Statistical significance was assessed by Mann–Whitney U test (** *p* < 0.01, NS: non-significant). (**D**) Boxplot of Shannon diversity between treatment groups. Statistical significance was assessed by Mann–Whitney U test. (* *p* < 0.05, NS: non-significant) (**E**) Principal coordinate analysis plot of Bray–Curtis distances of samples colored by treatment group. (**F**) Venn diagram of LEfSe analysis of pairwise comparisons between treatment groups. Overlapping regions indicate shared discriminant features between groups.

Previous findings have shown that GWI immune responses were associated with viral dysbiosis [10]. Therefore, we performed viral sequencing of the mice fecal pellets to assess changes in the composition of the virome between treatment groups. Major viral families identified included *Microviridae, Siphoviridae*, and *Myoviridae* across all treatment groups (Figure 2A). While there were no significant differences in *Microviridae* counts between treatment groups, AG treatment resulted in significantly decreased *Siphoviridae*, *Myoviridae*, and *Inoviridae* RPK counts (Figure 2B). AG treatment alone or in combination resulted in significantly decreased *Myoviridae* and *Inoviridae* counts. To assess the overall composition changes between treatment groups, we measured contig richness and Shannon diversity. The richness and Shannon diversity were significantly decreased in AG-treated mice compared to GWT mice (Figure 2C,D). Principle coordinate analysis using unweighted Bray–Curtis distances was used to compare compositional relationships between individual samples (Figure 2E). The virome of Control and GWT mice overlapped (PERMANOVA *p* = 0.088), while the GWT+AG and AG mice clustered distinctly from one another (PERMANOVA *p* = 0.002; *p* = 0.001). We next sought to identify viral features discriminant between treatment groups. We performed LEfSe analysis and identified significant overlap in contigs associated with Control and GWT groups with fewer overlapping features with the AG-treated groups (Figure 2F). This indicated that AG treatment specifically affects certain components of the virome (Supplementary Table S2). These results also indicated that AG treatment significantly alters the diversity and composition of the gut virome independent of the healthy no-exposed virome.

### 3.3. AG Treatment Improves the Integrity of Intestinal Tight Junction Proteins and Decreases GW Chemical-Induced Gut Leaching

**Figure 3:**
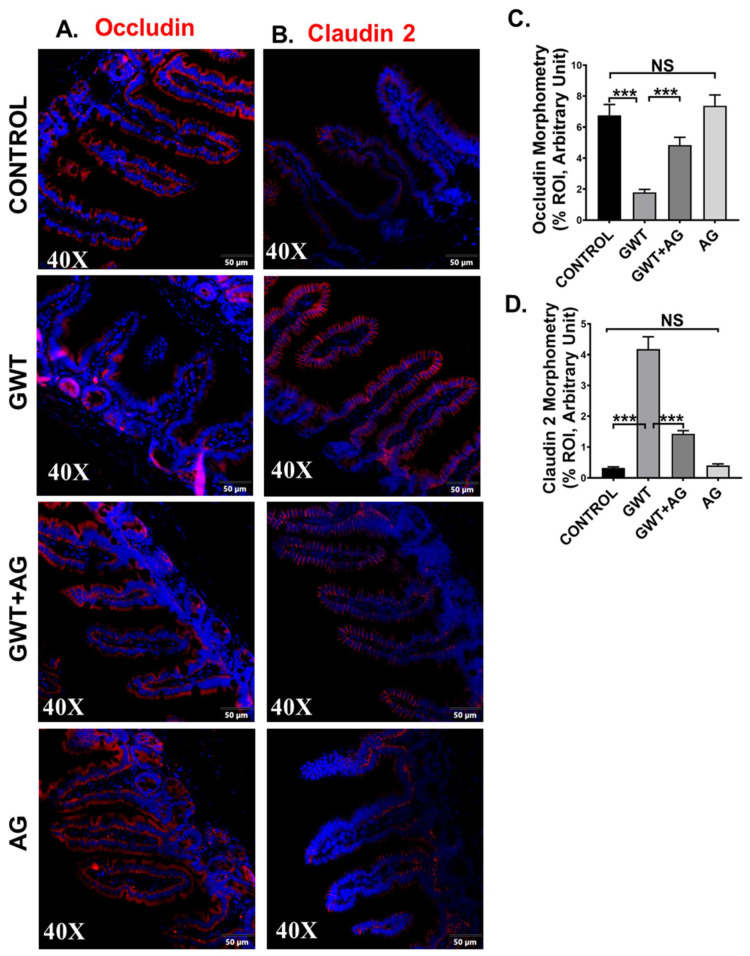
GW chemical exposure causes gut leakiness due to altered expression of intestinal tight junction proteins, improved by AG treatment. Formalin-fixed, paraffin-embedded 5 μm intestinal slices from CONTROL (mice treated with vehicle only), GWT (GW chemical exposed mice), GWT+AG (mice co-exposed to both GW chemicals and AG), and AG (mice treated with AG only) groups were used for immunofluorescence imaging. Representative immunofluorescence images depicting (**A**) Occludin and (**B**) Claudin-2 reactivity (red) in the small intestine, counterstained with DAPI (blue) of CONTROL, GWT, GWT+AG, and AG mice groups. Images were taken at 40× magnification. Morphometric analysis (calculated as %ROI) of (**C**) Occludin and (**D**) Claudin-2 immunoreactivity. *Y*-axis represents % positive immunoreactive area (% ROI) (*n* = 3; mean value taken from three separate microscopic fields) (*** *p* < 0.001, NS: non-significant). Data were represented as mean ± SEM and statistical significance was tested using unpaired *t*-test between the groups (* *p* < 0.05, ** *p* < 0.01, *** *p* < 0.001), followed by Bonferroni Dunn Post hoc corrections.

Tight junction (TJ) proteins are responsible for maintaining epithelial barrier integrity, which in turn is essential for regulating nutrient absorption, transport of selective content from the lumen, and blockage of pathogen entry [38,39]. In our previous studies, we showed that GW chemical exposure led to an altered expression of TJ proteins, where Claudin-2 expression was increased with a simultaneous decrease in Occludin expression, leading to gut leaching [7,11]. In this study, we wanted to show whether AG was able to revert the altered expression of TJ proteins in GW chemical-exposed mice. Results showed that Occludin expression significantly decreased (Figure 3A,C, *** *p* < 0.001), and Claudin-2 expression were significantly increased (Figure 3B,D, *** *p* < 0.001) in the GWT group when compared to the Control group consistent with our previous studies. However, Occludin expression was significantly increased (Figure 3A,C, *** *p* < 0.001) whereas Claudin-2 expression decreased significantly (Figure 3B,D, *** *p* < 0.001) in the mice group co-exposed to both GW chemicals and AG, compared to the GWT group alone as detected by immunofluorescence microscopy. No significant change in Occludin or Claudin-2 protein expression was observed between the vehicle-treated control, and the only AG-treated mice groups (Figure 3A–D, NS = non-significant). Principle coordinate analysis of the virome showed a significant difference in the beta diversity of mice with high Occludin levels (PERMANOVA *p* = 0.041) but did not show a significant relationship with Claudin-2 levels (PERMANOVA *p* = 0.188) (Supplementary Figure S1). The virome also showed specific trends when analyzed by immune marker levels. MaAsLin2 was used to determine associations between immune marker levels and viral counts. Results showed a negative correlation between Occludin levels and significant contigs and a positive trend between Claudin-2 levels and viral contigs (Supplementary Table S3). These results confirm that AG played a direct role in restoring gut barrier integrity of GW chemical-treated mice partly via its role in restoring the virome and its direct effect on the expression of TJ proteins. A decontam analysis of virome samples are included in Supplementary Figure S2.

### 3.4. AG Attenuated Toll-Like Receptor-Mediated Downstream Proinflammatory NF-κB Signaling Pathway Following Altered Gut Bacteriome-Virome

**Figure 4:**
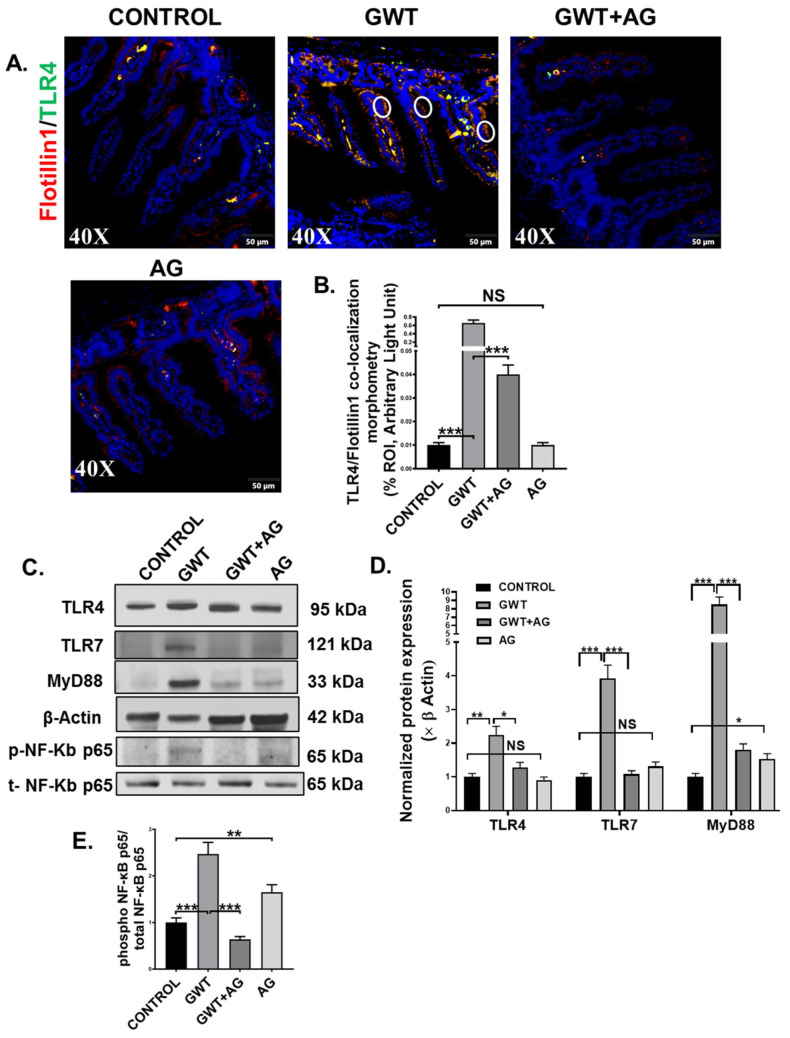
GW chemical-induced microbiome pattern alteration and gut leaching activate TLR4 and TLR7 pathways in the small intestine, leading to downstream inflammatory signaling activation. Representative immunofluorescence images depicting (**A**) Flotillin1 (red) and TLR4 (green) co-localization events in the small intestine, counterstained with DAPI (blue) of CONTROL, GWT, GWT+AG, and AG mice groups. Images were taken at 40× magnification. Co-localization was represented by the yellow dots and marked by white circles. (**B**) Morphometric analysis (calculated as %ROI) of Flotillin1-TLR4 co-localization events. *Y*-axis represents % positive immunoreactive area (% ROI) (*n* = 3; mean value taken from three separate microscopic fields) (*** *p* < 0.001, NS: non-significant). (**C**) Western blot images of TLR4, TLR7, MyD88, β-actin, phospho-NF-κB p65, and total-NF-κB p65 protein expression level obtained from intestinal tissue lysates. Lanes 1–4 represent CONTROL, GWT, GWT+AG, and AG mice groups, respectively. Densitometry analyses of (**D**) TLR4, TLR7, MyD88 immunoblots normalized against β-actin, and (**E**) phospho-NF-κB p65 immunoblot normalized against total-NF-κB p65 (* *p* < 0.05, ** *p* < 0.01, *** *p* < 0.001, NS: non-significant). Data were represented as mean ± SEM and statistical significance was tested using unpaired *t*-test between the groups (* *p* < 0.05, ** *p* < 0.01, *** *p* < 0.001), followed by Bonferroni Dunn Post hoc corrections.

A combined effect of the “leaky gut” phenotype and gut dysbiosis resulted due to GW chemical exposure can subsequently trigger the host’s TLR-mediated innate immune system [7,8,10]. Dual-labeling was performed on the intestine tissue sections to observe TLR4-Flotiilin1 co-localization events, which is a signature of TLR4 activation. Results showed a significantly increased co-localization of TLR4 and Flotillin1 (Figure 4A,B; *** *p* < 0.001) represented by yellow dots and encircled in white) in the epithelia of the small intestine compared to the Control group. However, this co-localization was significantly decreased in the GWT+AG group when compared to the GWT group alone (Figure 4A,B; *** *p* < 0.001). No significant difference of TLR4-Flotillin1 co-localization was observed between the Control, and only AG-treated mice groups (Figure 4A,B, NS = non-significant). This result indicated enhanced TLR4 trafficking to the lipid rafts of the intestinal epithelial region due to GW chemical-driven microbial dysbiosis and a leaky gut. We further confirmed our results by studying TLR4 protein expression level via Western blot analysis of small intestinal tissue lysates. Results showed an increased expression of TLR4 in the GWT group compared to the control group (Figure 4C,D, ** *p* < 0.01). Further, TLR4 total protein expression was decreased significantly in the GWT+AG group compared to the GWT group (Figure 4C,D, * *p* < 0.05). A significant difference in TLR4 expression was not found when the vehicle-treated Control group and only the AG-treated group were compared (Figure 4C,D, NS = non-significant). These results, when combined with TLR4-Flotillin1 co-localization events, confirmed activation and enhanced expression of TLR4 in the GW exposed mice and its modulation by AG. Next, we wanted to study whether AG-induced modulation of the virome in the GW chemical-treated mice and its downstream signaling could be mediated by the deactivation of TLRs known for virome sensing in the intestinal microenvironment. Western blot analysis showed that TLR7 protein expression was increased markedly in the GWT group when compared to Control while AG administration (GWT+AG group) (Figure 4C,D; *** *p* < 0.001) significantly decreased its expression. MyD88 is a known adaptor molecule essential for both TLR4 and TLR7 mediated downstream proinflammatory signaling pathways [40,41]. We observed that the MyD88 protein expression level was significantly enhanced in the GWT group compared to Control and AG administration significantly decreased MyD88 protein levels (Figure 4C,D; *** *p* < 0.001) and was able to restore the levels of these proteins to the control level (Figure 4C,D; * *p* < 0.05), (Figure 4C,D; NS = non-significant). Further, we wanted to investigate whether TLR-mediated downstream NF-κB signaling was activated. Indeed, Western blot analysis showed phosphor-NF-κB p65 expression (normalized against total-NF-κBp65) was markedly enhanced in the small intestine of the GWT group compared to the Control group (Figure 4C,E; *** *p* < 0.001). AG, being an NF-κB inhibitor, decreased phosphor-NF-κB p65 expression in the GWT and AG co-exposed mice compared to the GWT mice group (Figure 4C,E; *** *p* < 0.001).

### 3.5. AG Treatment Diminishes Both Intestinal and Systemic Inflammation in a Mouse Model of GWI

**Figure 5:**
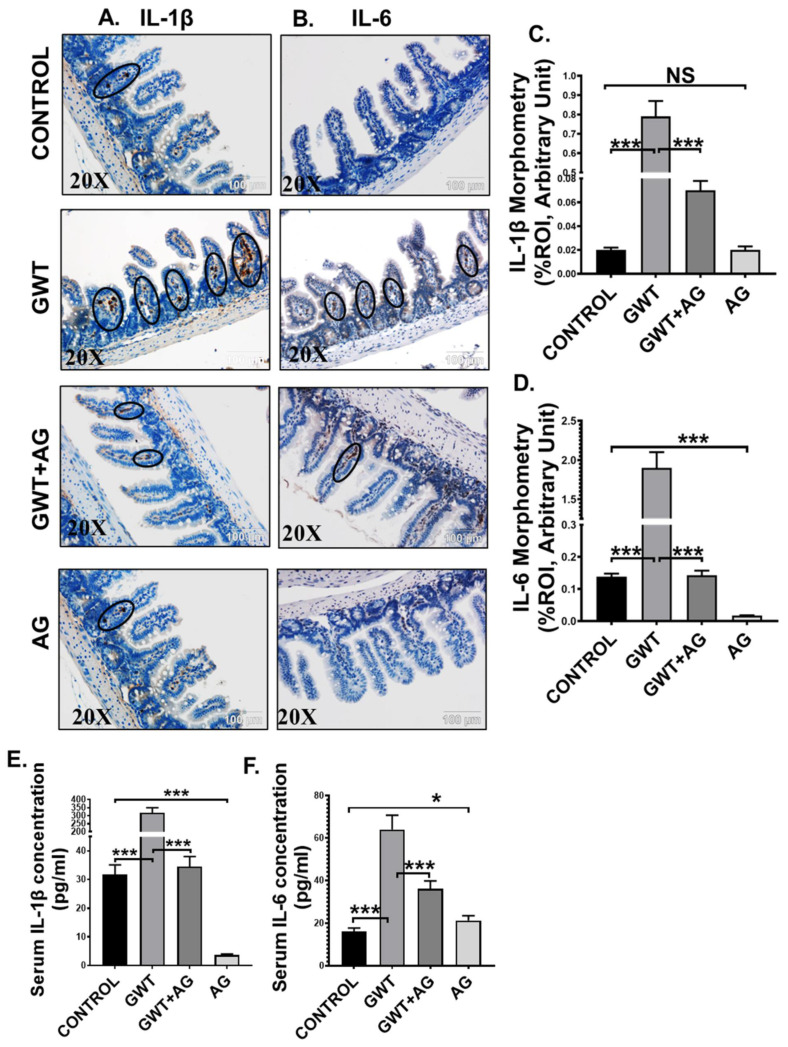
AG attenuates GW chemical-induced gut and systemic inflammation. Representative immunohistochemistry images of (**A**) IL-1β and (**B**) IL-6 immunoreactivity in the small intestine of CONTROL, GWT, GWT+AG, and AG mice groups. Images were captured in 20× magnification, and immunoreactivity was indicated by black arrows. Morphometric analysis (calculated as %ROI) of (**C**) IL-1β and (**D**) IL-6 immunoreactivity. *Y*-axis represents % positive immunoreactive area (% ROI) (*n* = 3; mean value taken from three separate microscopic fields) (*** *p* < 0.001, NS: non-significant). The serum concentration (pg/mL) of both (**E**) IL-1β and (**F**) IL-6 levels were measured in CONTROL, GWT, GWT+AG, and AG mice groups and plotted as bar graphs. Data were represented as mean ± SEM and statistical significance was tested using unpaired *t*-test between the groups (* *p* < 0.05, ** *p* < 0.01, *** *p* < 0.001), followed by Bonferroni Dunn Post hoc corrections.

Activation of TLR4/TLR7-MyD88-NF-κB signaling triggers the production of proinflammatory cytokines (IL-1β, IL-6), which are responsible for localized inflammation [42]. Using immunohistochemistry, we observed that immunoreactivity (observed as brown precipitation and marked inside black circles) of both IL-1β and IL-6 was markedly increased in the villi region of the small intestine of the GW chemical exposed group compared to the Control group (Figure 5A–D; *** *p* < 0.001). AG administration significantly lowered both IL-1β and IL-6 immunoreactivity in the intestines of the GWT+AG group compared to the GWT group (Figure 5A–D; *** *p* < 0.001). Notably, IL-6 expression was found to be significantly decreased in the only AG-treated group (Figure 5A–D; *** *p* < 0.001) compared to the Control group, but no significant difference was observed in the case of IL-1β expression (Figure 5A–D; NS = non-significant). We performed ELISA to check the serum level of both IL-1β and IL-6 cytokines. Results showed a significant 10-fold increase in IL-1β concentration and a 4-fold increase in IL-6 concentration in the serum obtained from GW chemical exposed groups compared to that of the Control group (Figure 5E,F; *** *p* < 0.001). The mice group co-exposed with both GW chemicals and AG had lower serum IL-1β and IL-6 levels compared to the GWT group (Figure 5E,F; *** *p* < 0.001). Interestingly, serum IL-1β concentration in the only AG-treated group was markedly lowered compared to the vehicle-treated Control group (Figure 5E: *** *p* < 0.001), but serum IL-6 level was found to be significantly increased in the only AG group than the Control group (Figure 5F; * *p* < 0.05). Principal coordinate analysis was employed to understand the relationship between IL-6 concentration and virome composition, with no significant difference being found between IL-6 concentrations (PERMANOVA *p* = 0.168) (Supplementary Figure S1 Mendeley reference link provided). When MaAsLin2 was used to determine associations between IL-6 concentrations and viral contig counts, we found a positive trend between the two (Supplementary Table S3, Mendeley reference link provided). This suggested that AG not only restored a healthy virome but also attenuated the systemic release of proinflammatory mediators, an anticipated cause for systemic chronicity of inflammation found in GWI.

### 3.6. AG Reverses GWI-Associated Altered Expression of Blood–Brain Barrier (BBB) Claudin-5 Protein and Microglial Activation

**Figure 6:**
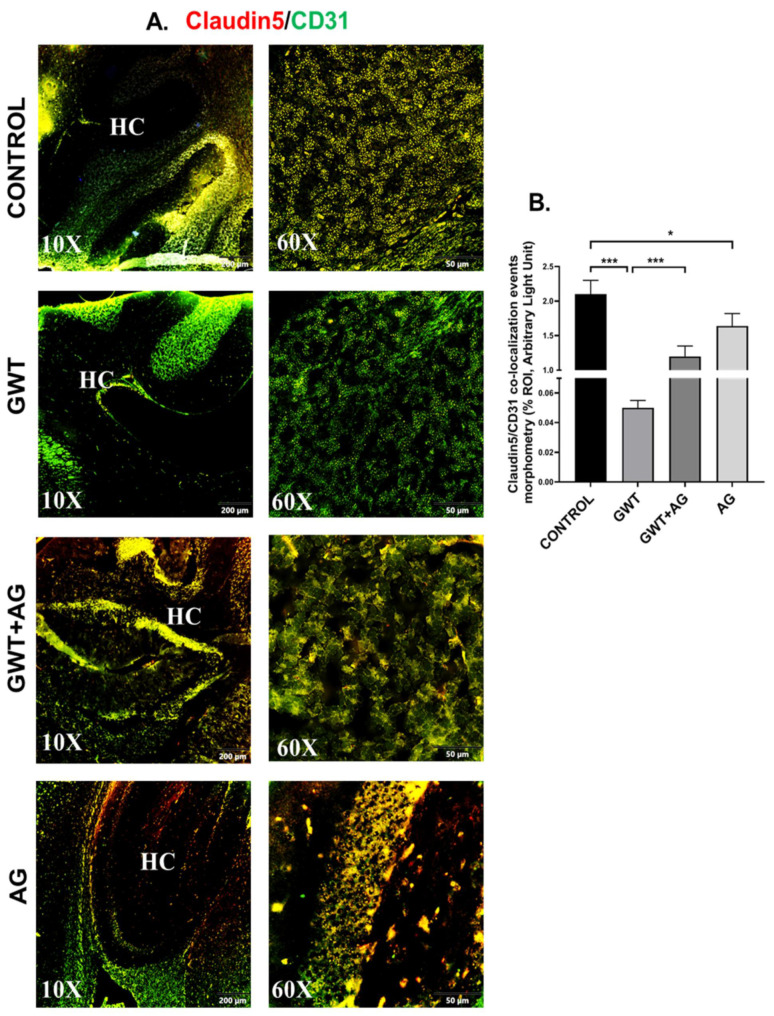
AG treatment improves GW chemical-induced Blood–Brain Barrier dysfunction. Representative immunofluorescence images depicting (**A**) Claudin-5 (red) and CD31 (green) co-localization events in the brain sections, counterstained with DAPI (blue) of CONTROL, GWT, GWT+AG, and AG mice groups. Images were taken at 10× and 60× magnification. Co-localization was represented by the yellow dots. The Hippocampus region is marked as HC. (**B**) Morphometric analysis (calculated as %ROI) of Claudin-5 and CD31 co-localization events. *Y*-axis represents % positive immunoreactive area (% ROI) (*n* = 3; mean value taken from three separate microscopic fields) (* *p* < 0.05, *** *p* < 0.001). Data were represented as mean ± SEM and statistical significance was tested using unpaired *t*-test between the groups (* *p* < 0.05, ** *p* < 0.01, *** *p* < 0.001), followed by Bonferroni Dunn Post hoc corrections.

Neurological disturbances resulted from various chemical exposure during warfare are one of the most commonly observed symptoms in GWI pathology [43,44]. The optimal functioning of BBB prevents the invasion of pathogens and toxins from the bloodstream to the brain. BBB selectively allows the entry of essential ions and molecules essential for the homeostatic condition of the Central nervous system [45,46]. We have shown previously that GW chemical treatment in mice was responsible for the loss of BBB integrity [9]. Consistent with our previous findings, we observed a significantly decreased co-localization of Claudin-5 (TJ Protein marker of BBB) and CD31 (brain endothelial cell marker) in the GWT group compared to the vehicle-treated Control group (Figure 6A,B; *** *p* < 0.001). However, AG treatment in GW chemical exposed mice significantly increased Claudin-5 and CD31 co-localization events (represented by yellow) in the GWT+AG group compared to the GWT group alone (Figure 6A,B; *** *p* < 0.001), referring to attenuation of BBB integrity loss. Analysis of the effects of Claudin-5 and CD31 concentrations on the virome composition yielded no significant difference in beta diversity over different concentrations (Supplementary Figure S1, Mendeley_Reference link provided). This is further supported by MaAsLin2 analysis finding no distinct correlation between Claudin-5 and CD31 levels and viral contig counts (Supplementary Table S3, Mendeley reference link provided).

**Figure 7:**
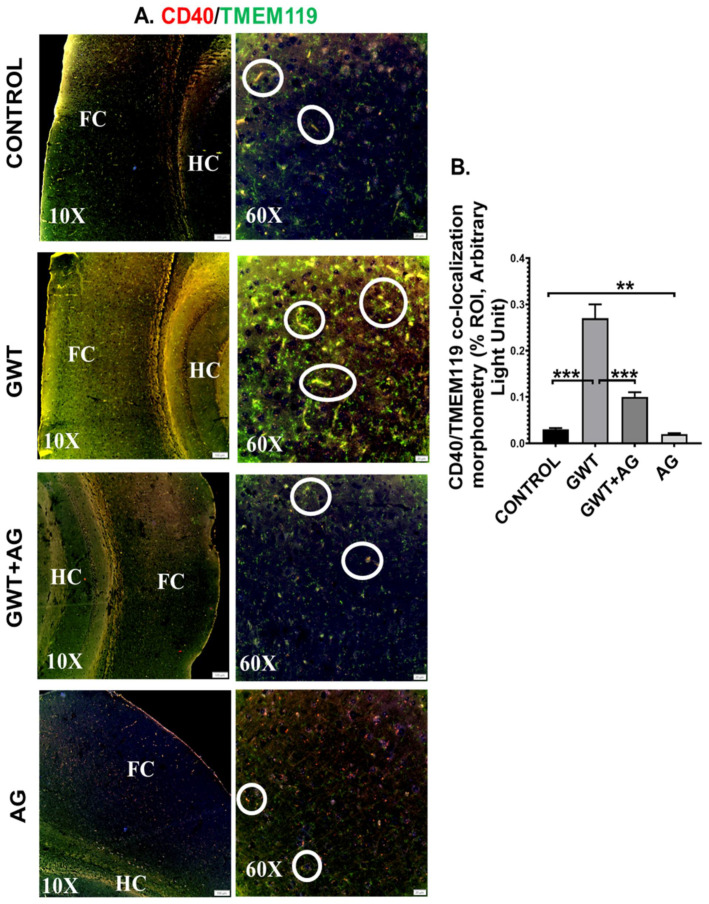
Microglial activation, caused by GW chemical treatment, is decreased by AG administration. Representative immunofluorescence images depicting (**A**) CD40 (red) and TMEM119 (green) co-localization events in the brain sections, counterstained with DAPI (blue) of CONTROL, GWT, GWT+AG, and AG mice groups. Images were taken at 10× and 60× magnification. Co-localization was represented by the yellow dots and marked by white circles. The Hippocampus region is marked as HC, whereas the frontal cortex is marked as FC. (**B**) Morphometric analysis (calculated as %ROI) of CD40 and TMEM119 co-localization events. *Y*-axis represents % positive immunoreactive area (% ROI) (*n* = 3; mean value taken from three separate microscopic fields) (** *p* < 0.01, *** *p* < 0.001). Data were represented as mean ± SEM and statistical significance was tested using unpaired *t*-test between the groups (* *p* < 0.05, ** *p* < 0.01, *** *p* < 0.001), followed by Bonferroni Dunn Post hoc corrections.

Systemic inflammation and dysfunctional BBB often result in the activation of Microglia, resident macrophages of the brain [47,48]. In our present study, microglial activation was observed in the mouse brain by performing dual-labeling of TMEM119 (microglia-specific activation marker) and CD40 (microglial cell surface marker). Significantly increased co-localization of TMEM119-CD40 (marked by yellow and encircled in white) was observed in the GWT group compared to the Control group (Figure 7A,B; *** *p* < 0.001). Conversely, TMEM119-CD40 co-localization was found to be significantly decreased in the AG and GW chemical co-exposed group compared to the GWT group alone (Figure 7A,B; *** *p* < 0.001), suggesting AG treatment had a direct effect in attenuating the active state of microglia. 

### 3.7. AG Attenuates Neuroinflammation and Enhanced BDNF Expression in a Mouse Model of GWI

**Figure 8:**
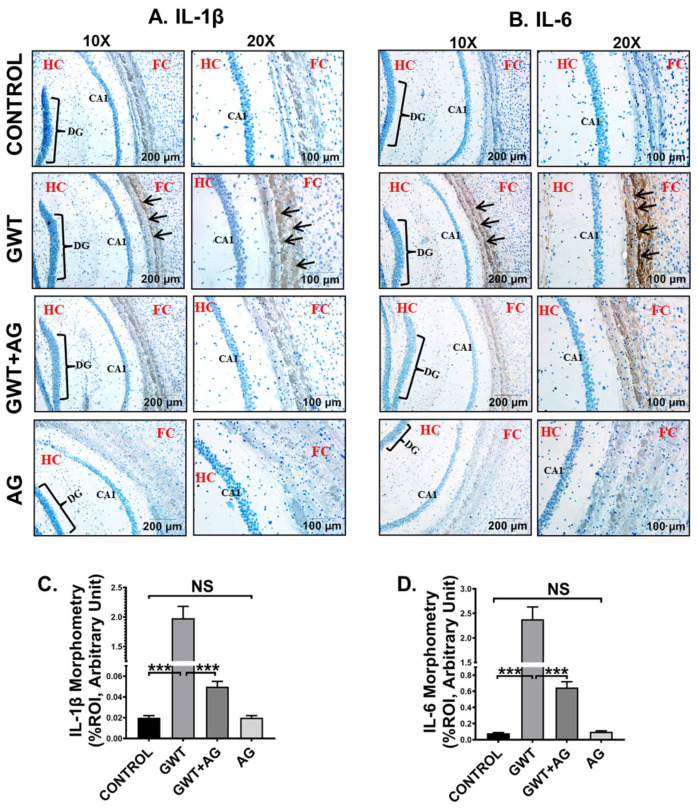
AG administration in GW chemical-treated mice decreases neuroinflammation. Representative immunohistochemistry images of (**A**) IL-1β and (**B**) IL-6 immunoreactivity in the brain sections of CONTROL, GWT, GWT+AG, and AG mice groups. Images were captured in 10× and 20× magnification, and immunoreactivity was indicated by black arrows. The Hippocampus region is marked as HC, the dentate gyrus area is marked as DG, the cornu ammonis area is marked as CA1, whereas the frontal cortex is marked as FC. Morphometric analysis (calculated as %ROI) of (**C**) IL-1β and (**D**) IL-6 immunoreactivity. *Y*-axis represents % positive immunoreactive area (% ROI) (*n* = 3; mean value taken from three separate microscopic fields) (*** *p* < 0.001, NS: non-significant). Data were represented as mean ± SEM and statistical significance was tested using unpaired *t*-test between the groups (* *p* < 0.05, ** *p* < 0.01, *** *p* < 0.001), followed by Bonferroni Dunn Post hoc corrections.

GW chemical-induced microglial activation triggers heightened neuroinflammation, a symptom that is strongly associated with GWI pathophysiology [49]. Immunohistochemistry results showed an increased expression of both proinflammatory cytokines IL-1β and IL-6 in the pre-frontal cortex area of the GWT mice group compared to the Control group (Figure 8A–D; *** *p* < 0.001). Neuroinflammation was observed to be significantly decreased in the brain sections of GWT+AG mice compared to GW chemical exposed mice (Figure 8A–D; *** *p* < 0.001), indicating the anti-inflammatory role of AG in the brain tissue. Furthermore, no significant expression of both IL-1β and IL-6 proinflammatory cytokines was observed between the Control group and only AG-treated group (Figure 8A–D; NS = non-significant), suggesting AG restored the normal levels of the cytokines mentioned above. 

**Figure 9:**
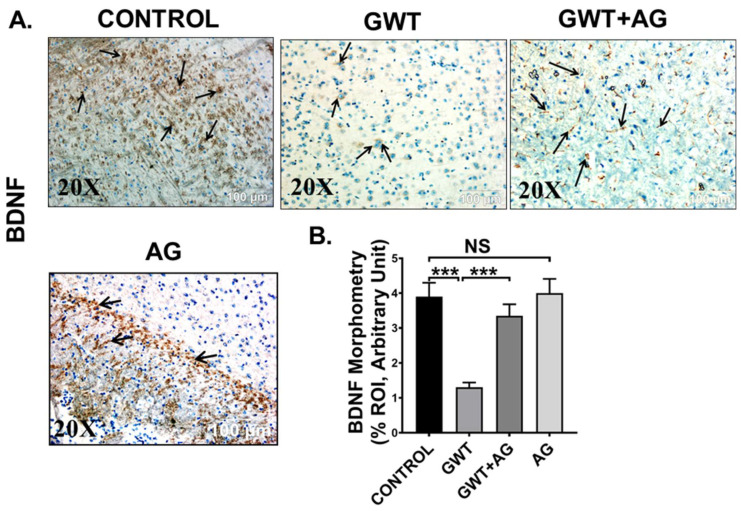
AG restores BDNF expression crucial for neurogenesis. Representative immunohistochemistry images of (**A**) BDNF immunoreactivity in the brain sections (the frontal cortex is focused) of CONTROL, GWT, GWT+AG, and AG mice groups. Images were captured in 20× magnification and immunoreactivity was indicated by black arrows. (**B**) Morphometric analysis (calculated as %ROI) of (BDNF immunoreactivity. *Y*-axis represents % positive immunoreactive area (% ROI) (*n* = 3; mean value taken from three separate microscopic fields) (*** *p* < 0.001, NS: non-significant). Data were represented as mean ± SEM and statistical significance was tested using unpaired t-test between the groups (* *p* < 0.05, ** *p* < 0.01, *** *p* < 0.001), followed by Bonferroni Dunn Post hoc corrections.

Microglial activation, together with exacerbated neuroinflammation leads to decreased production of BDNF, a protein that has a prominent role in synaptic plasticity and neuronal regeneration [50]. As observed by immunohistochemistry results, BDNF protein expression was markedly decreased in the frontal cortex area of GW chemical exposed mice compared to the Control mice, but the expression was significantly enhanced in the GWT+AG group (Figure 9A,B; *** *p* < 0.001). Significantly enough, BDNF expression was not significantly different between the Control group and only AG-treated group (Figure 9A,B; NS = non-significant), suggesting AG could restore the normal levels of BDNF. These results explicitly indicated the anti-inflammatory effects of AG in GWI-linked neuroinflammation and restoration of BDNF level by AG administration in GWI murine pathology.

### 3.8. AG Lessens TNFα Production from Reactive Mouse Microglial Cells, Concomitantly Protects Mouse Neuronal Cells from Extrinsic Apoptosis and Tau Hyperphosphorylation

**Figure 10:**
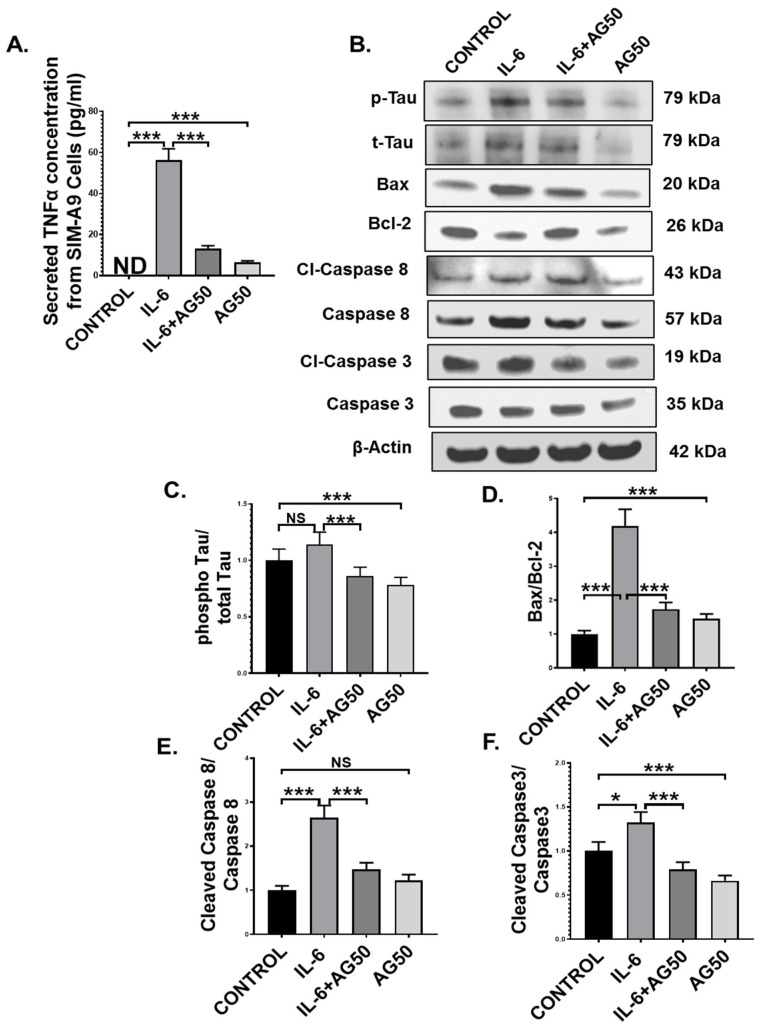
AG treatment decreased TNFα release from IL-6 primed SIM-A9 mouse microglial cells and MCM-induced extrinsic apoptosis, and tau phosphorylation in Neuro-2a mouse neuroblastoma cells. (**A**) The concentration (pg/mL) of TNFα level secreted from SIM-A9 cells treated with vehicle only (CONTROL), IL-6 only (IL-6), a combination of IL-6 and AG 50 µM (IL-6+AG50), and AG 50 µM only (AG50) as detected by ELISA using cell supernatants (ND = Not detected). Neuro-2a cells were treated with MCM collected from SIM-A9 cells, which were treated previously with vehicle only (CONTROL), IL-6 only (IL-6), a combination of IL-6 and AG 50 µM (IL-6+AG50), and AG 50 µM only (AG50). (**B**) Western blot images of p-Tau, t-Tau, BAX, Bcl-2, Cl-Caspase 8, Caspase 8, Cl-Caspase 3, Caspase 3 protein expression level obtained from Neuro-2a cell lysates. Lanes 1–4 represent CONTROL, IL-6, IL-6+AG50, and AG50 groups, respectively. Densitometry analyses of (**C**) phospho-Tau immunoblot normalized against total-Tau, (**D**) BAX immunoblot normalized against Bcl-2, (**E**) Cleaved Caspase 8 immunoblot normalized against Total Caspase 8 immunoblot, and (**F**) Cleaved Caspase 3 immunoblot normalized against Total Caspase 3 immunoblot (* *p* < 0.05, *** *p* < 0.001, NS: non-significant). Data were represented as mean ± SEM and statistical significance was tested using unpaired *t*-test between the groups (* *p* < 0.05, ** *p* < 0.01, *** *p* < 0.001), followed by Bonferroni Dunn Post hoc corrections.

Microglia, the resident macrophages of the central nervous system, turn into an active state when they encounter various external stimuli, which include cytokines, DAMPs, PAMPs, ROS, and neurotoxins [51]. Although activated microglia are responsible for mitigating external danger to the CNS, hyperreactive microglia contribute to various neuropathological conditions, including GWI [49]. In our study, we observed the systemic rise of the proinflammatory cytokine IL-6, a pivotal mediator of microglial activation, as well as microglial activation itself in vivo in the GWT group, both of which were decreased by AG treatment. This led us to hypothesize that perhaps, this IL-6 hypercytokinemia-led microglial activation may result in the release of TNFα, which in turn may act as an initiator of the extrinsic apoptotic pathway and tau hyperphosphorylation in neuronal cells. To prove our hypothesis, immortal mouse microglial SIM-A9 cells were treated with vehicle (CONTROL) only, IL-6 (IL-6) only, 50 µM AG (AG50) only, and a combination of both IL-6 and 50 µM AG (IL-6+AG50). IL-6 concentration (64 pg/mL) used for the in vitro experiment was kept the same as the in vivo ELISA result obtained for the GWT group. Indeed, Our ELISA result showed that TNFα secretion by the SIM-A9 cells was significantly increased in the only IL-6 stimulated group (IL-6) compared to both CONTROL and IL-6+AG50 groups, respectively (Figure 10A; *** *p* < 0.001). As expected, no TNFα was detected in the CONTROL group (Figure 10A: ND = Not detected). 

Next, we used microglia conditioned medium (MCM) obtained from SIM-A9 cells to treat mouse neuroblastoma Neuro-2a cells for 24 h. Western Blot results showed that pro-apoptotic protein (BAX: Bcl-2) expression was markedly higher in the IL-6 group compared to either CONTROL or the IL-6+AG50 group (Figure 10B,D; *** *p* < 0.001). Subsequently, both initiator caspase protein expression level (cleaved-Caspase 8 protein expression normalized against total Caspase 8 expression) and executioner caspase protein expression level (Cleaved Caspase 3 protein expression normalized against total caspase 3 expression) was found significantly increased in the IL-6 stimulated MCM treated neuronal cells when compared to both CONTROL and the IL-6+AG50 groups (Figure 10B,E,F; * *p* < 0.05, *** *p* < 0.001). Although pro-apoptotic protein (BAX: Bcl-2) expression and Cleaved Caspase 3 expression was significantly increased in the AG50 group compared to the CONTROL group, no significant change in expression level was observed for cleaved Caspase 8 (Figure 10B,D–F; NS = non-significant *** *p* < 0.001). In addition, phosho-Tau protein expression (normalized against total-Tau) was found to be increased, although non-significantly in the IL-6 group compared to the CONTROL group, a significant decrease in expression was observed in the IL-6+AG50 group when compared to only IL-6 primed group (Figure 10B,C; NS = non-significant *** *p* < 0.001). However, phospho-Tau expression was significantly decreased in the AG50 group in comparison to the CONTROL group as obtained by our Western Blot results (Figure 10B,D–F; *** *p* < 0.001). These results vividly proved our hypothesis and established a mechanistic role of AG in neutralizing reactive microgliosis as well as the neuroprotective role of AG from extrinsic apoptosis and GWI-associated Tau hyperphosphorylation in neurons.

## 4. Discussion

Gulf War Illness is a chronic multisymptomatic illness often characterized by a myriad of symptoms ranging from chronic fatigue, pain, gastrointestinal disturbances, and cognitive deficits [52]. Our present study shows profound broad-spectrum probiotic, anti-inflammatory, and neurotrophic effects of andrographolide (AG) in a mouse model of GWI. GWI is presented in the clinics, but a distinct management plan for its treatment remains elusive. Several preclinical studies have shown effective treatment modalities by using small molecule intermediates that target the gut microbiome, systemic inflammation, and/or anti-neuroinflammatory domains [53,54]. A recent spurt in trials of probiotics in attenuating the GWI symptoms has shown promise after our group established the role of the microbiome in blood–brain barrier dysfunction and neuroinflammation in the rodent models of GWI [7,9,10]. We report a novel role of the diterpenoid lactone AG in exerting a broad-spectrum effect on the gut bacteriome and virome, gastrointestinal inflammation, BBB dysfunction, microglial activation, and loss of neuronal regeneration via BDNF. 

We have shown previously that an altered microbiome in the host gut primarily because of the exposure to GW chemicals leads to a leaky gut, release of damage-associated molecular patterns, systemic increase in the proinflammatory cytokines such as IL-1β, and IL-6 [6]. We also showed that microbiome and virome alterations are strongly linked to BBB dysfunction and neuroinflammation thus ascribing a distinct role of the Gut–Brain axis in the pathology [10]. Thus, to tackle such broad pathological consequences arising from a pathway that connects multiple organ systems need a broad-spectrum small molecule that would tackle the Gut–Brain-Axis efficiently. Our results showed that AG administration to mice significantly increased probiotic bacterial species such as *Lachnospiraceae* and *Akkermansia*. Interestingly, both of these species have been found to be immensely helpful in maintaining robust gut and immune health [55,56,57]. Further, a rise in butyrogenic bacterial species *Bifidobacterium* following AG administration supports the probiotic role of AG probably via increasing the butyrate levels in the gut and the systemic circulation. The above increase in butyrate might also help in promoting anti-inflammatory events by binding to GPR109A and triggering a robust Treg response [58]. On the other hand, AG has been shown to be a strong inhibitor of NF-kB signaling thus blunting proinflammatory pathways [59]. These roles depicted above might act in concert to amplify the anti-inflammatory pathways thus attenuating DAMP release from the leaky gut. 

One of the important mediators of proinflammatory pathways is the virome itself [60]. We have shown that the altered virome plays an integral role in the Gut–Brain-axis related pathology in GWI [10]. AG administration not only restored the normal/healthy virome but significantly created a distinct virome pattern where the classes of *Myoviridae* and *Siphoviridae* were down-regulated, suggesting that the associated inflammatory pathways triggered by the altered virome, e.g., increased systemic levels of IL-6 may be decreased because of the virome-altering role of AG. Notably, an altered virome is also associated with NAFLD and ulcerative colitis pathology [61,62]. AG effects on the virome and subsequent proinflammatory pathways may be significantly effective in GI disturbances in general. AG’s role in virome modulation is novel and is in addition to the anti-viral role of this compound established in other studies [63]. GI disturbances and several other diseases that are chronic in nature exhibit a leaky gut via the alteration of tight junction proteins, namely, Occludin and Claudin-2 [64,65]. GWI has been found to harbor a leaky gut and a decreased expression of Occludin with a concomitant rise in protein levels of Claudin-2 [6]. AG administration significantly restored the normal balance of these proteins suggesting that it was effective in attenuating the leaky gut since there was a parallel decrease in IL-6 levels in the serum. Interestingly expression of either Claudin-2 or Occludinn is regulated by a separate mode of proteins and small molecule intermediates endogenously found in the intestinal epithelial cells. Vitamin D, transcription factors such as SNAIL and GRHL2 have been shown to be effectively modulating the expression of these proteins [35,66,67,68]. It would be interesting to study the mechanisms of AG in controlling the transcription factors both at the intestinal epithelium barrier and the BBB to know for certain the mechanisms involved in its effectiveness. In parallel, AG’s role in increasing the expression of bacteria such as *Akkermansia* and *Lachnospiraceae* may also have a role in preserving the gut barrier homeostasis since these bacteria have been shown to modulate the mucin layer in the gastrointestinal epithelium [35]. A link between the bacterial metabolites such as butyrate or others that can be released by *Akkermansia* may also induce transcriptional regulation of barrier proteins via GRHL2/SNAIL, but that is hypothetical at this point. 

AG was found to restore the virome diversity similar to healthy control mice in our study. The virome families of *Myoviridae* and *Siphoviridae* often can have TLR7 as their receptors for downstream signal modulations [69]. Our results of a mechanistic role of AG in downregulating the TLR7 and its adaptor molecule MyD88 suggest that the AG’s role in decreasing the abundance of *Siphoviridae* and *Myoviridae* may be playing a role in decreased expression of these receptors. However, a parallel role of AG-induced transcriptional regulation of both TLR7 and MyD88 cannot be ruled out especially when such regulations are known by histone deacetylases [70]. Interestingly AG did not alter TLR4 protein levels in the gut epithelia suggesting that the role of this broad-spectrum anti-viral may have a direct effect on the TLR7 pathway.

A leaky gut resulting from the loss of barrier integrity in the intestine often results in endotoxemia, activation of enteric glial cells, the release of nitric oxide, and localized inflammation in the epithelial cells of the intestine [12,71]. The gut epithelia can then secrete both IL-1β and IL-6, resulting in the significant increase of these proinflammatory mediators in the systemic circulation [72]. AG significantly decreased these mediators in the gut epithelia and in the systemic circulation, suggesting that the AG effect on the virome, attenuation of TLR7 based downstream signaling, and its effect in attenuating NF-kB might have resulted in decreased IL-1β and IL-6 protein levels in the epithelia and a subsequent release of the mediators in the portal circulation. This pans well for a significant decrease not only in the local milieu but also attenuates the parallel link to the brain since gut proinflammatory mediators will most likely cross the BBB and result in inflammation of the frontal cortex. AG-induced decrease in systemic levels of IL-1β and IL-6 is also proof that our hypothesis of AG attenuation of epithelial cell inflammation is well versed given that these cellular mediators can finally drain into the circulation. 

Having established the role of AG in attenuating gastrointestinal inflammation, we studied the effect of proinflammatory mediators and their likely effect on the BBB since the barrier is a principal component of the Gut Brain-Axis. Our results of AG-induced restoration of Claudin-5 expression in the brain endothelial cells in vivo suggest a significant role of AG in preserving this vital membrane integrity. Notably, we did not study the leaky membrane at the BBB as this study, coupled with the estimation of Claudin-5 levels, would have suggested that AG not only restored the protein levels of Claudin-5 but was effective in functionally restoring it. A focused study involving AG’s role in BBB function alone would definitely enhance the feasibility of the use of this natural compound in other diseases that are linked to BBB dysfunction. 

The BBB serves as a cohesive anatomical structure with defined functional roles that are linked to several cell types such as pericytes, microglia, astrocytes, and the neuron [45,73]. It serves as the entry point of circulatory mediators both responsible for normal physiological functions and immune system regulation in the brain tissue. The endothelial cells of the BBB have pronounced crosstalk with the above cell types and are often responsible for maintaining homeostasis [73]. Microglia and astrocytes play an important role in immune regulation, but many inflammatory phenotypes such as that seen in Alzheimer’s disease (AD) or Parkinsonism result from proinflammatory actions of these cell types [74,75]. For example, microglial activation may result in the release of proinflammatory cytokines such as IL-1β, MCP-1 that in turn can activate the surrounding astrocytes for roles that may perturb neurogenesis and synaptic plasticity [76,77]. GWI has been shown to affect microglial action, astrocyte activation, more importantly, resulting in decreased BDNF release, an important neurotrophic factor [11]. AG was found in our study to significantly decrease microglial activation thus causing a decreased release of local IL-1β and IL-6 in the brain microenvironment. The above result is very significant and is relevant to the attenuation of the proinflammatory signaling cascade that might have arisen because of the ability of AG to block microglial activation. Furthermore, this result also might pave the way for attenuating a decrease of BDNF release from the cells surrounding the microglia, namely, the neurons in GW chemical exposed mice. To show that the crosstalk amongst the various immune cell types, especially microglia and neurons, a mechanistic study needed to be performed that would ensure that AG-induced mitigation of proinflammatory pathways in the brain runs through its actions on the microglia. We collected the microglial secretome via harvesting the microglial conditioned medium (MCM) and subsequently incubated it with the neuronal cells with this medium. Our results of AG attenuating the phosphorylation of Tau, a clinical indicator of neurodegeneration and is clinically associated with AD, proved that the natural broad-spectrum compound could not only attenuate inflammation but could also decrease the accumulation of phosphorylated Tau via microglial deactivation. Strikingly, GWI veterans have been found to have significantly elevated levels of Tau antibodies that may have resulted from increased phosphorylation of Tau though the mechanisms remain unclear [78]. Nevertheless, phosphorylated Tau remains a clinical biomarker of neurodegeneration and collapse of the cognitive trail [79,80,81]. AG-induced decrease of phosphorylated Tau via microglial deactivation might be an important pathway for its anti-inflammatory function in the brain microenvironment. The decreased levels of apoptosis in the neuronal cells via the AG-treated microglia, induced by a parallel IL-6 mediated pathway, also bears proof that AG is significantly attenuating neuronal degeneration as a result of inflammation. 

In summary, we report a novel role of AG in regulating the Gut–Brain-Axis pathology in a mouse model of GWI. Though on a smaller scale, the in vivo result in this murine model definitely adds to a novel body of evidence that is specific to the broad-spectrum role of a natural compound that has been used in countries such as India and China for ages. This also bodes very well for a single drug approach to treat a plethora of chronic multisymptomatic illnesses such as chronic fatigue syndrome, fibromyalgia in addition to GWI. As we learn more about the role of the Gut–Brain Axis in the pathology of diseases, natural compounds with such broad-spectrum roles might be needed to mitigate the chronicity of the diseases mentioned above. Moreover, the usefulness of a drug that has very few side effects combined with a history of being used for generations may well fit for the quick bench to bedside strategies. However, more mechanistic studies need to be carried out in each component of the Gut–Brain Axis compartment to ensure the specific yet novel roles of this compound. Further, additional studies in a symptom persistence model that truly reflects the health state of the GW veteran need to be performed to show the therapeutic effect of andrographolide.

## 5. Conclusions

As supported by both in vivo and in vivo evidence obtained from the current study, the phytochemical andrographolide proved to be a potent therapeutic candidate for GWI-associated pathology and can be further examined by clinical trials to treat the GWI veterans.

## Data Availability

The data presented in this study are available on request from the corresponding author.

## Supplementary Materials

The following are available online at https://www.mdpi.com/article/10.3390/brainsci11070905/s1, Figure S1. (A) PCoA plot of viral Bray–Curtis distances colored by Occludin level (PERMANOVA *p* = 0.041). (B) PcoA plot of viral Bray–Curtis distances colored by Claudin-2 level (PERMANOVA *p* = 0.19). (C) PCoA plot of viral Bray–Curtis distances colored by IL-6 level (PERMANOVA *p* = 0.17). (D) PCoA plot of viral Bray–Curtis distances colored by Claudin-5 and CD31 levels (PERMANOVA *p* = 0.35), Figure S2. Decontam relative abundance of virome family collected from fecal pellets. Relative abundance of samples colored by decontam contaminant classification at a prevalence threshold of 0.3. Red indicates sample-specific values that were retained for analysis. Blue indicates values classified as contaminants that were removed, Table S1. Viral taxonomy of all contigs used in the study. Taxonomy retrieved from BLAST accession IDs using taxonomizr, Table S2. Viral features identified by LEfSe as discriminant by treatment group. Pairwise comparisons between treatment groups are shown in each tab, Table S3. Significant results were identified by MaAsLin2 for Occludin, IL-6, Claudin-2, and Claudin-5, and CD31 levels.
