# Supplementary figures and images for: Andrographolide Attenuates Gut-Brain-Axis Associated Pathology in Gulf War Illness by Modulating Bacteriome-Virome Associated Inflammation and Microglia-Neuron Proinflammatory Crosstalk
Source: Brain Sci. 2021 Jul 9;11(7):905. doi: 10.3390/brainsci11070905 (PMC8304847; doi:10.3390/brainsci11070905)

**Supplementary Fig. 1**

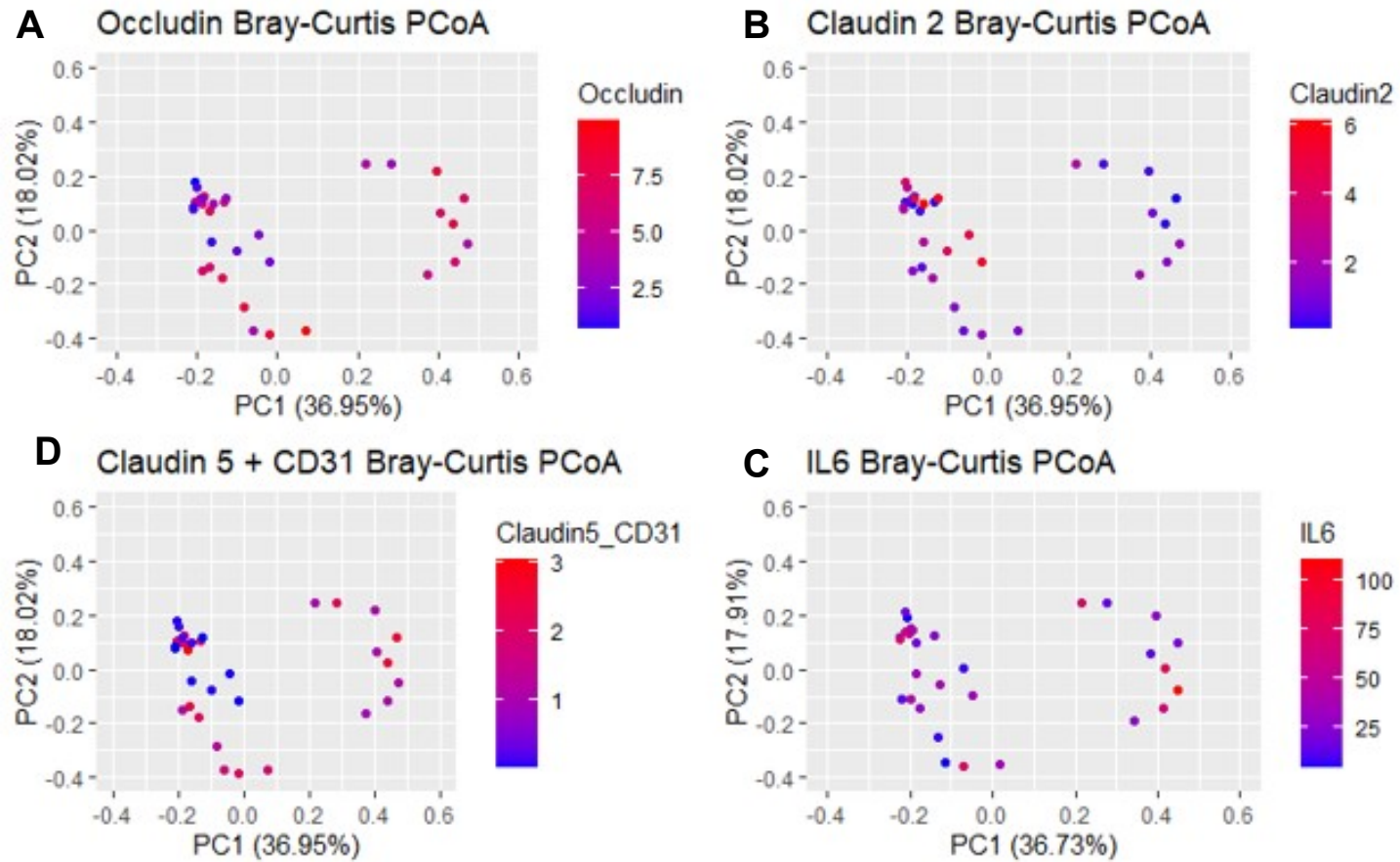

Supplementary Fig. 2

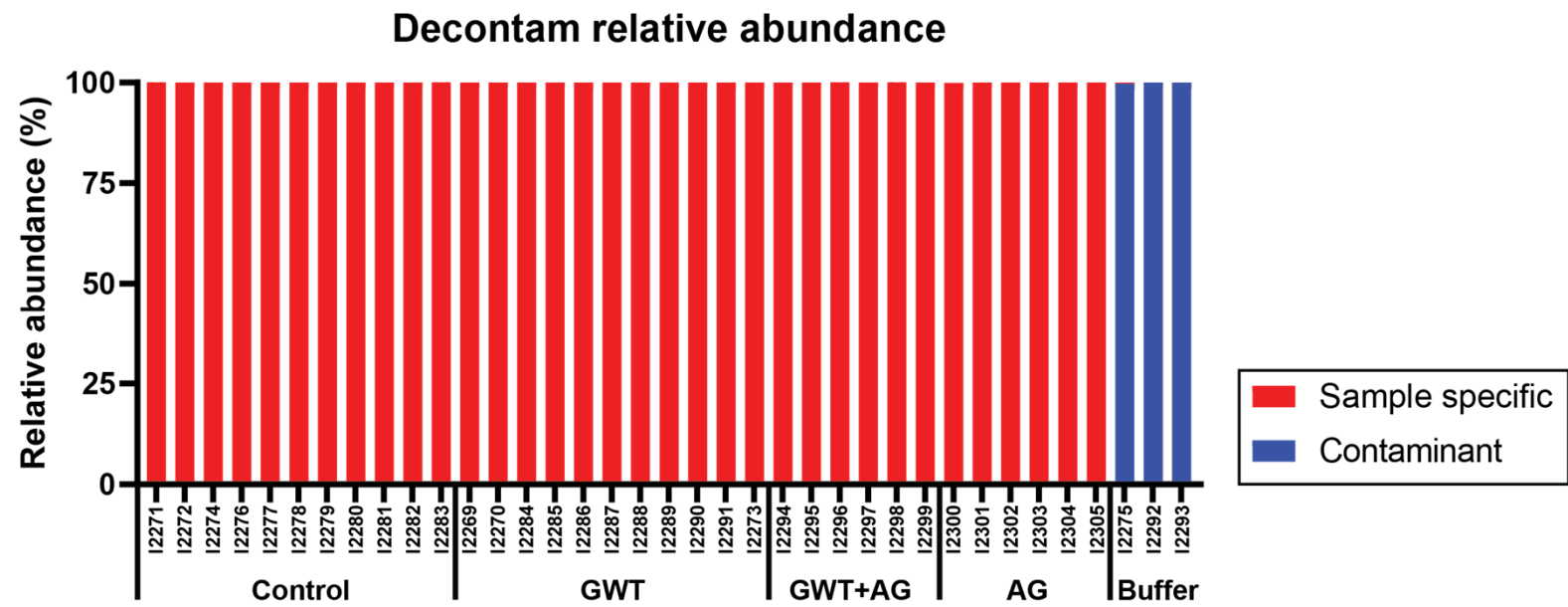

Supplement: Supplementary file 1 [file brainsci-11-00905-s001.zip › brainsci-1285377-supplmentary/brainsci-1285377-Figure S1 Figure S2.pdf]
